# Nowcasting Unemployment Rates with Google Searches: Evidence from the Visegrad Group Countries

**DOI:** 10.1371/journal.pone.0127084

**Published:** 2015-05-22

**Authors:** Jaroslav Pavlicek, Ladislav Kristoufek

**Affiliations:** 1 Institute of Information Theory and Automation, Academy of Sciences of the Czech Republic, Pod Vodarenskou vezi 4, Prague 8, 182 08, Czech Republic; 2 Institute of Economic Studies, Charles University, Opletalova 26, 110 00, Prague, Czech Republic; 3 Warwick Business School, University of Warwick, Coventry, West Midlands, CV4 7AL, United Kingdom; MIT, UNITED STATES

## Abstract

The online activity of Internet users has repeatedly been shown to provide a rich information set for various research fields. We focus on job-related searches on Google and their possible usefulness in the region of the Visegrad Group - the Czech Republic, Hungary, Poland and Slovakia. Even for rather small economies, the online searches of inhabitants can be successfully utilized for macroeconomic predictions. Specifically, we study unemployment rates and their interconnection with job-related searches. We show that Google searches enhance nowcasting models of unemployment rates for the Czech Republic and Hungary whereas for Poland and Slovakia, the results are mixed.

## Introduction

Online activity has become an inherent part of modern society and a way of living among its members. The Internet provides a vast amount of information to its users as well as aid and assistance in times of need. During the current financial crisis and the subsequent economic and production crises, most of the developed as well as developing economies are being hit by an economic downturn that is tightly connected with growing unemployment. Job loss can be a very traumatizing experience with long lasting impact on those who experience it. Seeking a new job then becomes an integral part of everyday life. In the current digitalized era, job seeking does not restrict itself to job offices because seekers (as well as potential employers) increasingly turn to the Internet as a source of information and new possibilities. As such, job seekers leave a digital track of their activity.

The analysis and examination of various patterns of online activity have become a fruitful branch of research in recent years with some exciting applications such as elections [1], investment allocation [2, 3], private consumption [4] and consumer behavior [5], future orientation [6], earnings announcements [7], spread of disease [8–12], and economics and finance [13–19]. In terms of unemployment and its possible examination utilizing the online activity of Internet users, there has been some research done in the area that focuses primarily on Google engine search queries. The first study focusing on the possible connection between Google search activity and unemployment rates in Germany shows the usefulness of adding search query data into models [20]. The subsequent research [21–23] analyzed the connection between queries and claims for unemployment benefits in the USA and the unemployment rate itself has also been studied [24, 25]. A job search activity index based on Google search data has even been developed [26]. Most of these studies focus on the US economy and its modeling, while other economies have been studied rather marginally [27, 28].

Here, we focus on the possible connection between job-related search queries using the Google search engine and the unemployment rate in countries of the so-called Visegrad Group (the Czech Republic, Hungary, Poland and Slovakia). Our contributions lay in the following. First, we focus on a set of countries that would be normally treated as marginal and that are thus not often studied. However, if claims are made for the utility of online search activity (and specifically Google searches), its efficiency should be shown not only for developed and well covered countries but also for the smaller ones, and the results might prove useful to all policy makers even in these types of regions. Second, we provide a careful step-by-step procedure for unemployment modeling, focusing not only on simple correlations but also on nowcasting with an out-of-sample analysis. Third, a cross-countries comparison is delivered that is rather unique given comparable studies that focus primarily on one specific country.

## Results

**Fig 1:**
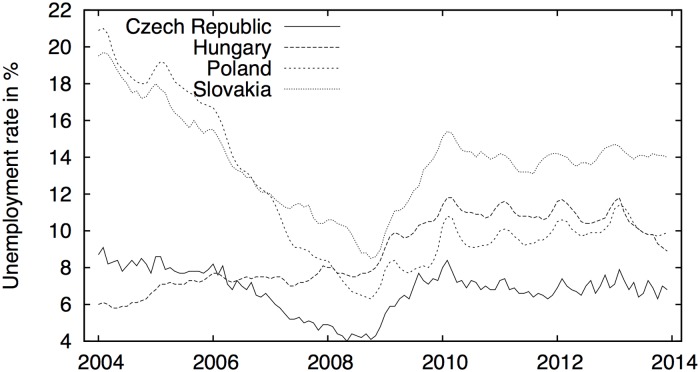
Unemployment rate in the Visegrad countries. The group of countries is evidently quite heterogenous in the unemployment rates. The Hungarian rate starts at the lowest level but increases stably during the whole period. The Czech rate begins at quite low levels and decreases up to the outbreak of the financial crisis when the rate surges up until 2010, after which it remains quite stable. The Polish and Slovakian rates commence at very high levels of unemployment, which decrease again up until the outbreak of the crisis, after which they change trends, similarly to the Czech rate.

The unemployment rates have undergone a quite heterogenous evolution in the analyzed countries (Fig 1). In the Czech Republic, the rate ranged between 4% and 9% between the years 2004 and 2013. Initially, there was a significant downward trend from 2004 to 2008, when the rate dropped from 9% to 4%. As the recession hit the Czech Republic in 2008, the rate started to increase to reach a new maximum of 8.5% in 2010. Since that time, the unemployment rate has fluctuated between 7% and 8.5%. The Hungarian unemployment rate steadily rose from the year 2004 to 2010, at which date it reached a new maximum of nearly 12%. After that point, the rate fluctuated for almost 3 years between 10.5% and 12% and started declining in 2013. Unemployment in Poland experienced a steady decline from the astronomical rate of nearly 22% in the 2004 to 6% in 2009. However, as the recession hit Poland, the unemployment rate began rising again. With some minor fluctuations, it smoothly increased to the current level of approximately 10%. In Slovakia, the unemployment rate appears to have a similar pattern as that in the Czech Republic, although on a different scale. In 2004, Slovakia had an unemployment rate of almost 20%. This rate linearly decreased to 8% in 2009. With the recession, the unemployment rate quickly escalated to 16%, and it has fluctuated around that point since. The differences between countries are well illustrated in the descriptives statistics provided in Table 1. Mainly for Poland and Slovakia, we observe wide fluctuations in time which are mirrored in higher variance and range of the unemployment rates. Even though the rates are close to being symmetric and show no strong excessive kurtosis, normality is rejected for all but Slovakia.

**Table 1:** Summary statistics. Jarque-Bera test with the null hypothesis of a symmetric distribution with no excess kurtosis is used here, *p*-values are reported in the brackets.

	Czech Rep.	Hungary	Poland	Slovakia
average	6.773	8.917	11.558	13.745
median	6.900	8.650	9.950	14.000
SD	1.179	1.872	3.987	2.491
minimum	4.000	5.800	6.300	8.500
maximum	9.100	11.800	21.000	19.700
skewness	-0.644	0.014	0.948	0.160
excess kurtosis	-0.207	-1.507	-0.419	-0.067
Jarque-Bera test	8.498	11.364	18.855	0.536
*p*-value	[< 0.05]	[< 0.01]	[< 0.01]	[> 0.10]
observations	120	120	120	120

**Fig 2:**
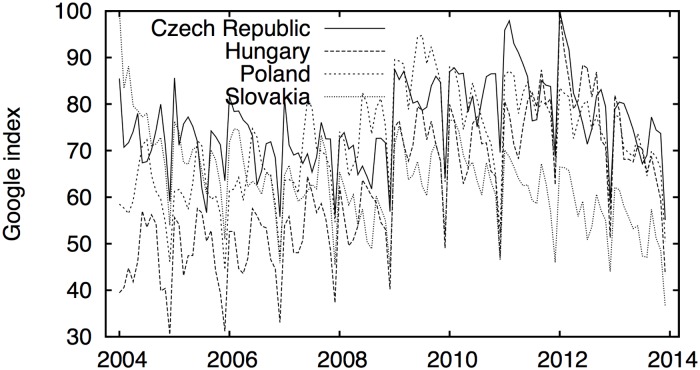
Google search queries for the job-related terms in the Visegrad countries. The patterns are again quite heterogenous, and the connection between the Google searches and the unemployment rates can be observed for the Czech and Hungarian rates. For the other two, the connection is not visible by the naked eye. Detailed treatment of the interconnections is given in the Results section of the text. Google data are registered trademarks of Google Inc., used with permission.

The evolution of Google searches is illustrated in Fig 2. There are evident seasonal patterns in all four series. Hungary is characterized by a quite regularly increasing trend in Google searches, whereas Slovakia shows the opposite, and the remaining two analyzed series remain quite stable over time. Although there appears to be a connection between Google searches and the unemployment rates for the Czech Republic and Hungary that is visible to the naked eye, we can hardly claim any relationship without a proper analysis. Information about the data collection and selection of Google search terms is provided in the Methods section.

### Basic relationship

As the initial step, we present the results of stationarity tests, which tell us whether we should analyze the original series or some of their transformations. In Table 2, we show the results of the ADF and KPSS tests (see the Methods section for more details) for the original as well as the logarithmic series and their first differences. The outcome is quite straightforward, as we do not reject unit roots for either of the original series (or their logarithmic transformation for the Google searches; we do not examine the logarithmic transformation for the unemployment time series as these are already in the percentage representation). Further testing, which is not reported here, shows no cointegration relationship between unemployment and the search query series, so we need to proceed with the first differences of the series. For most of the cases, we support the stationarity of the first differences. In the analysis, we further proceed with the first differences of the unemployment rate and the first logarithmic differences of the Google searches. We opt for this specification because the combination of percentage representation and logarithmic transformation allows for a straightforward interpretation as an elasticity, i.e., as a proportional relationship.

**Table 2:** Stationarity testing. Augmented Dickey-Fuller test (ADF) for a presence of unit root and KPSS test for stationarity are used. *, ** and *** stand for statistical significance at 10%, 5% and 1% levels, respectively. Number of lags for the tests is based on the Akaike Information Criterion (AIC) selection.

	Czech Rep.	Hungary	Poland	Slovakia
*ADF test*				
Unemployment	-1.6066	-1.78611	-2.6739*	-2.6438*
- first difference	-5.3860***	-4.5134***	-3.5267***	-4.2349***
Google	-0.6897	-1.3974	-2.1745	-0.8000
- logarithm	-0.6931	-1.1728	-2.3280	-0.3644
- difference	-11.4213***	-10.8293***	-11.1560***	-11.8463***
- logarithmic difference	-11.5094***	-10.9022***	-11.0750***	-11.7591***
*KPSS test*				
Unemployment	0.5399**	2.5995***	1.7946***	0.7507***
- first difference	0.1932	0.2848	0.6708**	0.5673**
Google	0.8294***	1.9059***	1.3281***	1.1640***
- logarithm	0.8193***	1.9375***	1.3596***	1.1737***
- difference	0.0889	0.1580	0.1358	0.1122
- logarithmic difference	0.0977	0.1480	0.1362	0.0967

For the very basic relationship between the unemployment rate and the intensity of job-related searches on Google, we study the equation

$$\begin{array}{c} \Delta {\text{UR}}_{t}=\alpha_{0}+\alpha_{1}\Delta \log{\left(\text{GI}\right)}_{t}+\varepsilon_{t} \end{array}$$ (1)

where ΔUR_*t*_ and Δlog(GI)_*t*_ represent the first difference of an unemployment rate at time *t* and the first logarithmic difference of the Google searches at time *t*, respectively, for a given country, and *ε*_*t*_ is an error term.

The elasticity between Google searches and the unemployment rate from Eq 1 is estimated at 0.686 (with the *p* = value of 0.008), 0.185 (0.125), 0.331 (0.216) and 0.606 (0.001) for the Czech Republic, Hungary, Poland and Slovakia, respectively, with the heteroskedasticity and autocorrelation consistent (HAC) standard errors. The proportional relationship thus varies across the analyzed countries, but it remains positive for all four countries and statistically significant for two out of the four (at the 1% significance level). Specifically, the relationship is very strong for the Czech Republic and Slovakia, with values above 0.6. This result shows that the changes in the unemployment rate are well projected into the online search queries for vacancies and job-related terms. Studying the connection between these two variables thus appears to be promising and worth further utilization and investigation.

### Nowcasting

Macroeconomic time series, such as unemployment rates, have a special property that is not present for financial series or other series in natural sciences—they are available with a pronounced lag. This lag occurs due to data processing and collection, which usually take several months; even after this period, there are sometimes corrections to the reported values. This characteristic makes a series that is available immediately without any lag and that is strongly correlated with the variable of interest very useful for forecasting the present value of the variable without waiting several months. This type of forecasting of the present is usually referred to as “nowcasting”.

In the previous section, we showed that Google searches for job-related terms are related to the unemployment rate, which makes search queries potentially useful for the nowcasting of unemployment. As a nowcasting model, we consider

$$\begin{array}{c} \Delta {\text{UR}}_{t}=\beta_{0}+\sum_{i=3}^{L}\beta_{i}\Delta {\text{UR}}_{t-i}+\sum_{j=0}^{L}\gamma_{j}\Delta \log{\left(\text{GI}\right)}_{t-j}+\varepsilon_{t} \end{array}$$ (2)

where the unemployment rate is assumed to be available with a three month lag (please refer to the Methods section for more details about the data collection). We again consider the differenced series due to the stationarity issues discussed above. As a base model, we use the model specification in Eq 2 without the Google terms so that the competing model is defined as

$$\begin{array}{c} \Delta {\text{UR}}_{t}=\delta_{0}+\sum_{i=3}^{L}\delta_{i}\Delta {\text{UR}}_{t-i}+\nu_{t}. \end{array}$$ (3)

For both models, we consider a maximum lag *L* which we set as *L* = 3, 6, 12. This way, we are able to comment on the quality of models with regards to the amount of information taken into consideration. The upper bound is set to 12 months as the unemployment series are usually strongly cyclical.

The results of the nowcasting models are summarized in Table 3. In the table, we show the adjusted *R* ^2^ (${\bar{R}}^{2}$) as a measure of the models’ quality controlling for the number of explanatory variables. We observe that for all countries, the inclusion of the Google series strongly enhances the model. The most promising results are reported for the Czech Republic where the models improve strongly regardless the number of lags taken into consideration. For the other three countries, we observe that the base models with 3 and 6 lags are very weak, even reaching negative values of ${\bar{R}}^{2}$. A strong seasonal (annual) pattern in the unemployment rates is thus visible here. The Google series are thus evidently useful for the in-sample modeling of the series, which is supported by a statistical significance of the online searches for all countries and regardless the maximum lag used. However, it is the out-of-sample performance that eventually matters.

**Table 3:** Nowcasting summary (in-sample). The whole analyzed period 01/2004-12/2013 is covered here. Model in Eq 2 is used here with varying maximum lag *L*. Joint significance of variables is a simple *F*-test based on heteroskedasticity and autocorrelation consistent (HAC) standard errors (*p*-values are reported in the brackets). Adjusted coefficient of determination ${\bar{R}}^{2}$ controls for the number of independent variables used in the model.

		Czech Rep.	Hungary	Poland	Slovakia
Δ*u*_*t*−*i*_ significance	*L* = 3	48.115	0.965	0.290	11.424
		[< 0.01]	[> 0.10]	[> 0.10]	[< 0.01]
	*L* = 6	20.559	0.863	7.188	6.336
		[< 0.01]	[> 0.10]	[< 0.01]	[< 0.01]
	*L* = 12	10.361	2.120	8.332	1.871
		[< 0.01]	[< 0.05]	[< 0.01]	[< 0.10]
Δlog*GI*_*t*−*i*_ significance	*L* = 3	9.284	2.945	7.472	5.454
		[< 0.01]	[< 0.05]	[< 0.01]	[< 0.01]
	*L* = 6	5.944	3.815	5.929	3.638
		[< 0.01]	[< 0.01]	[< 0.01]	[< 0.01]
	*L* = 12	7.685	3.574	2.525	7.448
		[< 0.01]	[< 0.01]	[< 0.01]	[< 0.01]
${\bar{R}}^{2}$, without Google	*L* = 3	0.177	0.022	-0.009	0.044
	*L* = 6	0.288	-0.003	-0.016	0.044
	*L* = 12	0.280	0.249	0.467	0.161
${\bar{R}}^{2}$, with Google	*L* = 3	0.318	0.076	0.144	0.163
	*L* = 6	0.367	0.118	0.328	0.205
	*L* = 12	0.407	0.418	0.552	0.406

We divide the analyzed period into two—a training (fitting) period and a nowcasting period. The model parameters are fitted on the data between 01/2004 and 12/2011 (96 observations). Nowcasting performance is then evaluated on the series between 01/2012 and 12/2013 (24 observations). The “Google model” (Eq 2) is compared to the base model (Eq 3) using the Diebold-Mariano test [29] (see the Methods section for more details). In Table 4, the resulting statistics are summarized. For the Czech Republic, the model using Google searches is on average outperforming the base model for each lag selection. For the maximum lag of 3 and 12 months, the difference is statistically significant. Similar results are reported for Hungary, for which the Google specification outperforms the base model for all lag selections as well. The difference is statistically significant for lags up to 3 and 6 here. For Poland, we find statistical significance only for the maximum lag of 6 months, and for Slovakia, the base model even outclasses the “Google model”. The results are thus quite diverse.

**Table 4:** Nowcasting summary (out-of-sample). The period between 01/2004 and 12/2011 is used for model fitting and the rest of the period between 01/2012 and 12/2013 is used for the forecasting comparison. Diebold-Mariano test described in the Methods section compares the “Google model” defined in Eq 2 to the base model defined in Eq 3 with a null hypothesis of no difference of forecasting accuracy versus the alternative of the “Google model” being more accurate.

		Czech Rep.	Hungary	Poland	Slovakia
Diebold-Mariano test	*L* = 3	1.326	2.2895	0.6467	-0.744
		[< 0.10]	[< 0.05]	[> 0.10]	[> 0.10]
	*L* = 6	0.979	1.5425	1.635	-1.203
		[> 0.10]	[< 0.10]	[< 0.10]	[> 0.10]
	*L* = 12	2.229	0.3312	-0.146	-4.021
		[< 0.05]	[> 0.10]	[> 0.10]	[> 0.10]

## Discussion

Data showing the online activity of Internet users has proven useful in various fields. Nowcasting the unemployment rate is one of these fields. Contrary to the prevailing trend in the literature focusing on well-developed (Western) countries, we have utilized job-related Google searches in the Visegrad Group countries, i.e., the Czech Republic, Hungary, Poland and Slovakia. Although data availability and Internet utilization might not be as widespread in this region as one would expect for developed countries, we have shown that, in fact, online searches provide a strong foundation for unemployment modeling.

In summary, we have shown that the basic dynamics of Google searches for job-related terms closely follow the unemployment rates. Further, we have utilized this idea to successfully nowcast the unemployment rates using the current and lagged values of Google searches. Our findings indicate that the information left online by Internet users can be easily utilized even for small or medium countries such as those of the Visegrad Group. However, this is true mainly for the Czech Republic and Hungary but much less so for Poland and especially Slovakia. Even though one of the reasons might be a different level of Internet penetration in the regions, we speculate that such diversity is caused by different customs and specifically international mobility in the analyzed countries. The Czech and Hungarian nationals usually do not move for work inside their country and even less so internationally. However, this is not the case for the Polish and Slovak citizens which are willing to move for work abroad. This is also reflected in the Google searches. For the job-related searches in Poland, one of the topical keywords is “gumtree” which relates to the UK advertising website, which well reflects willingness of the Polish nationals to seek job abroad. In a similar way, the topical keywords for Slovakia include “práce” and “prace” which are the Czech equivalents to the Slovak “práca” and “praca”. This again mirrors frequent moves of the Slovaks to the Czech Republic in their search for work. The international mobility of the Polish and Slovaks thus has a strong influence on the informative value of the Google searches and their usefulness for the unemployment modeling.

## Methods

### Data

The monthly unemployment data for the Czech Republic, Hungary, Poland and Slovakia have been obtained from the Eurostat database (http://ec.europa.eu/eurostat/). The basis for the unemployment measurement among EU countries is the EU Labour Force Survey (EU LFS)—a continuous and harmonized household survey that, in accordance with EU legislation, is conducted in each member state. The monthly data from Eurostat are estimates based on the results of EU LFS. Because there are no legal obligations that the EU countries deliver monthly data, these data are often interpolated/extrapolated using national surveys or registered unemployment data.

Eurostat defines an unemployed person as someone aged between 15 and 74 without work during the reference week who is available to start working within two weeks and who has actively sought employment at some time during the last four weeks. In our analysis, we use the general (both sexes, 15–74 years old), raw (not seasonally adjusted) unemployment rate. We use these data because we do not know the method used to make the seasonal adjustment and because the Google data are also not seasonally adjusted.

The Google search queries data have been downloaded from the Google Trends webpage (http://www.google.com/trends/). As the languages of the studied countries differ, we have looked for various terms. As Czech, Polish and Slovakian are all Slavonic languages, the searched words are very similar or even the same. For Czech, we searched for “práce” and “prace” (i.e. both with and without diacritics), for Polish “praca” and for Slovakian “práca” and “praca” (again both with and without diacritics) but also the Czech “práce” and “prace”, which turn out to be very frequently searched for by Slovakians. For Hungarian, we used the terms “állás” and “munka”. These all are equivalents for the English “job” and “work”. Other related words have not passed throught the Google threshold or only incomplete series are available. As the Google Trends engine allows to compare up to five terms for a given setting (in our case a country and a time frame), we can use more series for each country. In the cases of more searched queries (the Czech Republic, Hungary and Slovakia), we sum the series together and rescale them to 100. This can be done as if multiple series with the same spatial and temporal characteristics are obtained from the engine, these share a common scale.

The weekly series obtained from the Google Trends site have been transformed to monthly series on the basis of the number of days in the month. All series, both the unemployment rate and the Google searches, are studied between January 2004 and December 2013 (120 observations). The dataset is provided in the S1 Dataset.

### Stationarity

A stochastic process {*x*_*t*_} is stationary if for every collection of time indices 1 ≤ *t*_1_ < *t*_2_ < *t*_*m*_, the joint probability distribution of (*x*_*t*_1__, *x*_*t*_2__, …, *x*_*t*_*m*__) is the same as the joint probability distribution of (*x*_*t*_1+*h*__, *x*_*t*_2+*h*__, …, *x*_*t*_*m*+*h*__) for all integers *h* ≥ 1 [30]. To test for stationarity, we utilize the Augmented Dickey-Fuller (ADF) test [31] and the KPSS test [32]. The tests have opposite null hypotheses and thereby provide a complementary pair, which is commonly used for stationarity testing.

In the ADF procedure [31], the OLS regression is run on

$$\begin{array}{c} \Delta x_{t}=\alpha_{0}+\theta x_{t-1}+\gamma t+\Delta x_{t-1}+\Delta x_{t-2}+\cdots +\Delta x_{t-p}+\varepsilon_{t} \end{array}$$

to perform the test, where *α*_0_ and *γt* are an intercept and a time trend, respectively, and *p* represents the lag order. The null hypothesis under which the series contains a unit root is found for

$$\begin{array}{c} H_{0}:\theta =0 \end{array}$$

against the alternative

$$\begin{array}{c} H_{A}:\theta <0. \end{array}$$

The ADF test statistics are then computed as the usual *t*-statistics, which, however, follow a more complicated distribution under the null hypothesis.

The null hypothesis of the KPSS test [32] is opposite to that of the ADF test, i.e., the KPSS test has the null hypothesis of stationarity. The test is based on the OLS regression of the series {*x*_*t*_}:

$$\begin{array}{c} x_{t}=\alpha_{0}+\gamma t+k\sum_{i=0}^{t}\xi_{i}+\varepsilon_{t} \end{array}$$

where *α*_0_ and *γt* again represent an intercept and a time trend, respectively, and *ξ*_*i*_ are independent and identically distributed random variables with zero mean and a unit variance. The null hypothesis of stationarity is found for

$$\begin{array}{c} H_{0}:k=0 \end{array}$$

against the alternative

$$\begin{array}{c} H_{A}:k\neq 0. \end{array}$$

The KPSS test statistic is defined as

$$\begin{array}{c} KPSS=\frac{\sum_{t=1}^{n}S_{t}^{2}}{n^{2}{\hat{\omega }}_{T}^{2}} \end{array}$$

where *S*_*t*_ is the partial sum of the residuals

$$\begin{array}{c} S_{t}=\sum_{i=1}^{t}{\hat{\varepsilon }}_{i} \end{array}$$

and ${\hat{\omega }}_{T}^{2}$ is an estimator of the spectral density at frequency zero.

### Nowcasting accuracy

To compare the forecasting accuracy of the proposed models, we utilize the Diebold-Mariano test [29] based on absolute errors. An absolute error is simply defined as *a*_*i*_ = ∣*f*_*i*_ − *y*_*i*_∣ where *f*_*i*_ stands for a nowcast value and *y*_*i*_ is an observed real value. We do not use also popular squared errors here as these are usually applied to magnify higher errors. However, the nowcasting errors in our case are always lower than unity which makes the squared errors counterintuitive so that we avoid using them. Diebold and Mariano [29] propose a test to compare the predictive accuracy of two competing forecasts. Let ${\left\{\varepsilon_{t}^{1}\right\}}_{t_{0}}^{T}$ and ${\left\{\varepsilon_{t}^{2}\right\}}_{t_{0}}^{T}$ be the sequences of forecast error losses from two competing forecasting measures by a particular loss function (absolute errors *a*_*i*_ in our case). The null and alternative hypotheses are then stated as

$$\begin{array}{c} H_{0}:𝔼{\left\{\varepsilon_{t}^{1}\right\}}_{t_{0}}^{T}=𝔼{\left\{\varepsilon_{t}^{2}\right\}}_{t_{0}}^{T} \end{array}$$

$$\begin{array}{c} H_{A}:𝔼{\left\{\varepsilon_{t}^{1}\right\}}_{t_{0}}^{T}>𝔼{\left\{\varepsilon_{t}^{2}\right\}}_{t_{0}}^{T}. \end{array}$$

The Diebold-Mariano test assesses the accuracy based on the loss differential

$$\begin{array}{c} d_{t}={\left\{\varepsilon_{t}^{1}\right\}}_{t_{0}}^{T}-{\left\{\varepsilon_{t}^{2}\right\}}_{t_{0}}^{T} \end{array}$$

which is equal to zero under the null hypothesis. The Diebold-Mariano statistic is then

$$\begin{array}{c} S=\frac{\bar{d}}{\sqrt{{\hat{LRV}}_{\bar{d}}/T}} \end{array}$$

where $\bar{d}$ is the mean loss differential and ${\hat{LRV}}_{\bar{d}}$ is a consistent estimate of the asymptotic (long-run) variance of $\sqrt{T}\bar{d}$ defined

$$\begin{array}{c} LRV_{\bar{d}}=\gamma_{0}+2\sum_{j=1}^{\infty }\gamma_{j},\;\;\gamma_{j}=\text{cov}\left(d_{t},d_{t-j}\right). \end{array}$$

Under the null hypothesis, the testing statistic goes to a standard normal distribution so that $S\overset{A}{∼}N(0,1)$[29].

## Supporting Information

S1 Dataset (CSV)
